# Video-assisted thoracoscopic surgery under non-intubated spontaneous breathing anesthesia using laryngeal mask

**DOI:** 10.1186/1749-8090-10-S1-A272

**Published:** 2015-12-16

**Authors:** Premjithlal Bhaskaran, Antonios Katsipoulakis, Francesca Caliandro, Niall McGonigle, Nikolaos Anastasiou

**Affiliations:** 1 Department of Cardiothoracic Surgery, Harefield Hospital, Middlesex UB9 6JH, Royal Brompton & Harefield NHS Foundation Trust, UK; 2 Department of Cardiovascular Surgery, Hammersmith Hospital, Imperial College, London W12 0HS, UK; 3 Departments of Thoracic Surgery, Anesthesia and Postoperative Intensive Care, General Oncology Hospital of Kifissia "Agioi Anargyroi", Kaliftaki, P.C. 14564, Kifissia, Athens, Greece

## Background/Introduction

During the past 20 years, video-assisted thoracoscopic surgery (VATS) has been an important minimally invasive tool. In order to further reduce its invasiveness, non-intubated spontaneous breathing general anesthesia via a laryngeal mask (LMA) was used in a variety of thoracic procedures. The objective of this study is to evaluate the safety and feasibility of this advantageous technique.

## Aims/Objectives

From March 2013 till now, 23 patients with lung or pleural disease were managed by VATS under spontaneous breathing general anesthesia with LMA without using muscle relaxants.

## Method

**Table 1:** Clinical characteristics of the patients

Mean Age (years)	53.5
Age Range (years)	20-87
Male/Female	9/7
Weight (kg)	56-100
BMI (kg/m2)	<30
ASA class	I-II

**Table 2:** Type of LMA used

Type of LMA	Patients
LMA ProSeal	21
LMA Fastrach	2

**Table 3:** Type of thoracic procedure

Procedure	Patients
Pleural biopsy	2
Lung biopsy	4
Pulmonary nodule excision	4
Pericardial window	3
Multiloculated empyema debridement	2
Pneumothorax	8

## Results

The mean operative time was 40 minutes (range, 15-90 minutes). The values of lower oxygen saturation and peak end-tidal carbon dioxide tension were 95% and 50 mmHg, respectively. No mask displacement occurred. No conversion to endotracheal anesthesia was required, whereas one patient required conversion to mini thoracotomy. The level of technical feasibility was excellent in 12 cases and good in 11 cases. Mortality as well as morbidity rates were zero. Mean postoperative stay was 2.6 days.

## Discussion/Conclusion

It seems that VATS is safe and feasible under non-intubated spontaneous breathing anesthesia with LMA. A confident manipulation of lung parenchyma is allowed preventing from cough, pain, or panic attack described for awake epidural anesthesia, as well as avoiding the risks of tracheal intubation and mechanical ventilation.

